# Supplementary material for: A versatile genetic engineering toolkit for E. coli based on CRISPR-prime editing
Source: Nat Commun. 2021 Sep 1;12:5206. doi: 10.1038/s41467-021-25541-3 (PMC8410854; doi:10.1038/s41467-021-25541-3)
Supplement: Supplementary file 1 — Supplementary Information [file 41467_2021_25541_MOESM1_ESM.pdf]

Supplementary information for

A versatile genetic engineering toolkit for *E. coli* based on CRISPR-prime editing

Yaojun Tong[1,2,*], Tue S. Jørgensen[1], Christopher M. Whitford[1], Tilmann Weber[1,*], Sang Yup Lee[1,3,*]

[1]The Novo Nordisk Foundation Center for Biosustainability, Technical University of Denmark, 2800 Kgs. Lyngby, Denmark.

[2]State Key Laboratory of Microbial Metabolism, School of Life Sciences and Biotechnology, Shanghai Jiao Tong University (SJTU), Shanghai 200240, China.

[3]Department of Chemical and Biomolecular Engineering, BioProcess Engineering Research Center, BioInformatics Research Center, Institute for the BioCentury, Korea Advanced Institute of Science and Technology (KAIST), Daejeon 34141, Republic of Korea.

*To whom correspondence should be addressed yaojun.tong@sjtu.edu.cn (Y.T.), tiwe@biosustain.dtu.dk (T.W.), or leesy@kaist.ac.kr (S.Y.L.).

This file contains:

Supplementary note 1

Supplementary Tables 1-7

Supplementary Figures 1-6

Supplementary note 1

A second nick does not increase the editing efficiency of CRISPR-Prime Editing system in *E. coli*

It has been reported that the editing efficiency of the reverse transcriptase-Cas9 H840A nickase (Cas9n)-mediated targeted prime editing in some mammalian cells[1] and plant cells[2] can be increased by introduction of a second nick to the complementary strand within around 100 bp from the site of the PEgRNA-induced nick. However, it also increases NHEJ-mediated indel formation because Cas9 nickase with paired sgRNAs within ~200 bp can introduce targeted DSBs[3,4]. As most bacteria do not possess a complete NHEJ system[5-7], it makes the DSB a lethal event if no homology-directed repair (HDR) template is provided. It was thus reasoned that it is impossible to apply the strategy of introducing a close nick in the complementary strand in the NHEJ-deficient bacteria, like *E. coli*. To evaluate our hypothesis, we introduced another plasmid to deliver the designed complementary strand nicking sgRNA (nsgRNA). Two approaches for the secondary nick introduction were tested: nicking the non-edited strand within ~200 bp from the first nick (CRISPR-PE3) and nicking the non-edited strand only after the first nicked strand (the complementary strand) has been edited (CRISPR-PE3b). We designed editing events accordingly both in the plasmid-based system and the chromosome. As expected, for all three designed chromosomal DNA engineering and one plasmid DNA deletion of CRISPR-Prime Editing, almost no visible colonies were observed after the second nick was introduced (Supplementary Fig. 4).

Supplementary Table 1. Plasmids and strains involved in this study

Plasmid	Background	Reference
pdCas9-bacteria	*E. coli* codon optimized dCas9 is under control by a tetracycline inducible promoter; CamR; p15A ori	Addgene #44249[8]
pgRNA-bacteria	sgRNA transcript carrying plasmid, under control by a constitutive promoter J23119; AmpR; ColE1 ori	Addgene #44251[8]
pCDF-b1	SmR; CloDF13 ori	Millipore, US
pCDF-GFPplus	A fast folding GFP variant GFP+ is cloned into pCDF-b1 under control by a constitutive promoter	This study
pCRISPR-PE-bacteria	pdCas9- bacteria is used as the backbone. An *E. coli* codon optimized fusion protein of Cas9n-linker-M-MLV2 is cloned into pdCas9- bacteria by replacing the dCas9. The fusion protein is under control by a tetracycline inducible promoter.	This study
pPEgRNA	The 20 bp spacer was removed from pgRNA-bacteria. Therefore, this plasmid carries only a sgRNA scaffold without a spacer.	This study
pVRb20_992	KanR; pSC101 ori; carrying a cassette of PEcf20_992-sfGFP	Addgene #49714[9]
pnsgRNA	An sgRNA transcript cassette from pgRNA-bacteria was inserted to replace the PEcf20_992-sfGFP cassette	This study
pVRb_PEgRNA_312Gdel	pVRb_PEgRNA carries a 20 nt spacer targeting GFP coding gene, and a 3 prime extension for deleting the 312G, the length of both the reverse transcription template and the primer binding sequence is 13 bp	This study
pPEgRNA_GFP_TAAin	pPEgRNA carries a 20 nt spacer targeting GFP coding gene, and a 3 prime extension for inserting a stop codon TAA, the length of both the reverse transcription template and the primer binding sequence is 13 bp	This study
pPEgRNA_GFP_TAAin_T4	The same as pPEgRNA_GFP_TAAin, except that the length of the reverse transcription template is 4 bp	This study
pPEgRNA_GFP_TAAin_T5	The same as pPEgRNA_GFP_TAAin, except that the length of the reverse transcription template is 5 bp	This study

pPEgRNA_GFP_TAAin_T8	The same as pPEgRNA_GFP_TAAin, except that the length of the reverse transcription template is 8 bp	This study
pPEgRNA_GFP_TAAin_T20	The same as pPEgRNA_GFP_TAAin, except that the length of the reverse transcription template is 20 bp	This study
pPEgRNA_GFP_TAAin_PBS5	The same as pPEgRNA_GFP_TAAin, except that the length of the primer binding sequence is 5 bp	This study
pPEgRNA_GFP_TAAin_PBS8	The same as pPEgRNA_GFP_TAAin, except that the length of the primer binding sequence is 8 bp	This study
pPEgRNA_GFP_TAAin_PBS17	The same as pPEgRNA_GFP_TAAin, except that the length of the primer binding sequence is 17 bp	This study
pPEgRNA_GFP_T198A	pPEgRNA carries a 20 nt spacer targeting GFP coding gene, and a 3 prime extension for 198T to 198A substitution within the GFP coding gene, the length of both the reverse transcription template and the primer binding sequence is 13 bp	This study
pPEgRNA_GFP_T196C_T198C	pPEgRNA carries a 20 nt spacer targeting GFP coding gene, and a 3 prime extension for 196T，198T to 196C, 198C substitutions within the GFP coding gene, the length of both the reverse transcription template and the primer binding sequence is 13 bp	This study
pPEgRNA_GFP_198Tdel	pPEgRNA carries a 20 nt spacer targeting GFP coding gene, and a 3 prime extension for 198T deletion within the GFP coding gene, the length of both the reverse transcription template and the primer binding sequence is 13 bp	This study
pPEgRNA_GFP_combo	pPEgRNA carries a 20 nt spacer targeting GFP coding gene, and a 3 prime extension for 198T to 198A substitution, TAA insertion, and 199G deletion within the GFP coding gene, the length of both the reverse transcription template and the primer binding sequence is 13 bp	This study
pPEgRNA_GFP_12in	pPEgRNA carries a 20 nt spacer targeting GFP coding gene, and a 3 prime extension for insertion of a 12 bp fragment within the GFP coding gene, the length of both the reverse transcription template and the primer binding sequence is 13 bp	This study
pPEgRNA_GFP_18in	pPEgRNA carries a 20 nt spacer targeting GFP coding gene, and a 3 prime extension for insertion of a 18 bp fragment within the GFP coding gene, the length of both the reverse transcription template and the primer binding sequence is 13 bp	This study

pPEgRNA_GFP_33in	pPEgRNA carries a 20 nt spacer targeting GFP coding gene, and a 3 prime extension for insertion of a 33 bp fragment within the GFP coding gene, the length of both the reverse transcription template and the primer binding sequence is 13 bp	This study
pPEgRNA_GFP_10del	pPEgRNA carries a 20 nt spacer targeting GFP coding gene, and a 3 prime extension for deletion of a 10 bp fragment within the GFP coding gene, the length of both the reverse transcription template and the primer binding sequence is 13 bp	This study
pPEgRNA_GFP_23del	pPEgRNA carries a 20 nt spacer targeting GFP coding gene, and a 3 prime extension for deletion of a 23 bp fragment within the GFP coding gene, the length of both the reverse transcription template and the primer binding sequence is 13 bp	This study
pPEgRNA_GFP_36del	pPEgRNA carries a 20 nt spacer targeting GFP coding gene, and a 3 prime extension for deletion of a 36 bp fragment within the GFP coding gene, the length of both the reverse transcription template and the primer binding sequence is 13 bp	This study
pPEgRNA_GFP_49del	pPEgRNA carries a 20 nt spacer targeting GFP coding gene, and a 3 prime extension for deletion of a 49 bp fragment within the GFP coding gene, the length of both the reverse transcription template and the primer binding sequence is 13 bp	This study
pPEgRNA_GFP_97del	pPEgRNA carries a 20 nt spacer targeting GFP coding gene, and a 3 prime extension for deletion of a 97 bp fragment within the GFP coding gene, the length of both the reverse transcription template and the primer binding sequence is 13 bp	This study
pPEgRNA_lacZ_TAGin	pPEgRNA carries a 20 nt spacer targeting *lacZ* gene, and a 3 prime extension for TAG insertion into the *lacZ* gene for a stop codon introduction, the length for the reverse transcription template and the primer binding sequence is 16 bp and 13 bp, respectively	This study
pPEgRNA_lacZ_CGdel	pPEgRNA carries a 20 nt spacer targeting *lacZ* gene, and a 3 prime extension for CG deletion in the *lacZ* gene for a stop codon introduction, the length for the reverse transcription template and the primer binding sequence is 18 bp and 14 bp, respectively	This study
pPEgRNA_lacZ_GTtoTAsub	pPEgRNA carries a 20 nt spacer targeting *lacZ* gene, and a 3 prime extension for GT to TA substitution in the *lacZ* gene for a stop codon introduction, the length of both the reverse transcription template and the primer binding sequence is 14 bp	This study
pPEgRNA_galK_TAAin	pPEgRNA carries a 20 nt spacer targeting *galK* gene, and a 3 prime extension for TAG insertion into the *galK* gene for a stop codon introduction, the length for the reverse transcription template and the primer binding sequence is 16 bp and 13 bp, respectively	This study

pnsgRNA_GFP_TAAin	pnsgRNA carries a 20 nt spacer that pairs with pPEgRNA_GFP_TAAin to introduce the second nick	This study
pnsgRNA_lacZ_TAGin	pnsgRNA carries a 20 nt spacer that pairs with pPEgRNA_lacZ_TAGin to introduce the second nick	This study
pnsgRNA_lacZ_CGdel	pnsgRNA carries a 20 nt spacer that pairs with pPEgRNA_lacZ_CGdel to introduce the second nick	This study
pnsgRNA_lacZ_GTtoTAsub	pnsgRNA carries a 20 nt spacer that pairs with pPEgRNA_lacZ_GTtoTAsub to introduce the second nick	This study
**Strain**	**Background**	**Reference**
Escherichia coli DH10β (DH10B)	str. K F- mcrA Δ(mrr-hsdRMS-mcrBC) φ80lacZΔM15 ΔlacX74 recA1 endA1 araD139 Δ (ara, leu)7697 galU galK λ- rpsL nupG /pMON14272 / pMON7124. Whole-genome sequenced for parental strain characterization. The sequencing data can be found under SRA Accession: SRR15371477	Thermo Fisher Scientific, US
Escherichia coli MG1655	Str. K F-, lambda-, rph-1. Whole-genome sequenced for parental strain characterization. The sequencing data can be found under SRA Accession: SRR15371770	Maintained in lab
PE0001	*E. coli* DH10β carries pCDF-GFPplus	This study
PE0002	*E. coli* DH10β carries pCRISPR-PE-bacteria	This study
PE0003	*E. coli* DH10β carries pCRISPR-PE-bacteria and pCDF-GFPplus	This study
PE0004	*E. coli* DH10β carries pCRISPR-PE-bacteria, pCDF-GFPplus, and pPEgRNA	This study
PE0005	*E. coli* DH10β carries pCRISPR-PE-bacteria, pCDF-GFPplus, and pPEgRNA_GFP_TAAin. Whole-genome sequenced for mutation analysis. The sequencing data can be found under SRA Accession: SRR15371494	This study
PE0006	*E. coli* DH10β carries pCRISPR-PE-bacteria, pCDF-GFPplus, and pPEgRNA_GFP_TAAin_T4	This study

PE0007	*E. coli* DH10β carries pCRISPR-PE-bacteria, pCDF-GFPplus, and pPEgRNA_GFP_TAAin_T5	This study
PE0008	*E. coli* DH10β carries pCRISPR-PE-bacteria, pCDF-GFPplus, and pPEgRNA_GFP_TAAin_T8	This study
PE0009	*E. coli* DH10β carries pCRISPR-PE-bacteria, pCDF-GFPplus, and pPEgRNA_GFP_TAAin_T20	This study
PE0010	*E. coli* DH10β carries pCRISPR-PE-bacteria, pCDF-GFPplus, and pPEgRNA_GFP_TAAin_PBS5	This study
PE0011	*E. coli* DH10β carries pCRISPR-PE-bacteria, pCDF-GFPplus, and pPEgRNA_GFP_TAAin_PBS8	This study
PE0012	*E. coli* DH10β carries pCRISPR-PE-bacteria, pCDF-GFPplus, and pPEgRNA_GFP_TAAin_PBS17	This study
PE0013	*E. coli* DH10β carries pCRISPR-PE-bacteria, pCDF-GFPplus, and pPEgRNA_GFP_T198A. Clone #1. Whole-genome sequenced for mutation analysis. The sequencing data can be found under SRA Accession: SRR15371482	This study
PE0014	*E. coli* DH10β carries pCRISPR-PE-bacteria, pCDF-GFPplus, and pPEgRNA_GFP_T198A. Clone #2. Whole-genome sequenced for mutation analysis. The sequencing data can be found under SRA Accession: SRR15371480	This study
PE0015	*E. coli* DH10β carries pCRISPR-PE-bacteria, pCDF-GFPplus, and pPEgRNA_GFP_T196C_T198C. Whole-genome sequenced for mutation analysis. The sequencing data can be found under SRA Accession: SRR15371479	This study
PE0016	*E. coli* DH10β carries pCRISPR-PE-bacteria, pCDF-GFPplus, and pPEgRNA_GFP_198Tdel. Whole-genome sequenced for mutation analysis. The sequencing data can be found under SRA Accession: SRR15371493	This study
PE0017	*E. coli* DH10β carries pCRISPR-PE-bacteria, pCDF-GFPplus, and pPEgRNA_GFP_combo. Whole-genome sequenced for mutation analysis. The sequencing data can be found under SRA Accession: SRR15371478	This study
PE0018	*E. coli* DH10β carries pCRISPR-PE-bacteria, pCDF-GFPplus, and pPEgRNA_GFP_12in	This study

PE0019	*E. coli* DH10β carries pCRISPR-PE-bacteria, pCDF-GFPplus, and pPEgRNA_GFP_18in	This study
PE0020	*E. coli* DH10β carries pCRISPR-PE-bacteria, pCDF-GFPplus, and pPEgRNA_GFP_33in	This study
PE0021	*E. coli* DH10β carries pCRISPR-PE-bacteria, pCDF-GFPplus, and pPEgRNA_GFP_10del	This study
PE0022	*E. coli* DH10β carries pCRISPR-PE-bacteria, pCDF-GFPplus, and pPEgRNA_GFP_23del	This study
PE0023	*E. coli* DH10β carries pCRISPR-PE-bacteria, pCDF-GFPplus, and pPEgRNA_GFP_36del	This study
PE0024	*E. coli* DH10β carries pCRISPR-PE-bacteria, pCDF-GFPplus, and pPEgRNA_GFP_49del	This study
PE0025	*E. coli* DH10β carries pCRISPR-PE-bacteria, pCDF-GFPplus, and pPEgRNA_GFP_97del	This study
PE0026	*E. coli* MG1655 carries pCRISPR-PE-bacteria and pPEgRNA_lacZ_TAGin. Whole-genome sequenced for mutation analysis. The sequencing data can be found under SRA Accession: SRR15371774	This study
PE0027	*E. coli* MG1655 carries pCRISPR-PE-bacteria and pPEgRNA_lacZ_CGdel. Whole-genome sequenced for mutation analysis. The sequencing data can be found under SRA Accession: SRR15371773	This study
PE0028	*E. coli* MG1655 carries pCRISPR-PE-bacteria and pPEgRNA_lacZ_GTtoTAsub. Whole-genome sequenced for mutation analysis. The sequencing data can be found under SRA Accession: SRR15371772	This study
PE0029	*E. coli* MG1655 carries pCRISPR-PE-bacteria, pPEgRNA_galK_TAAin. Whole-genome sequenced for mutation analysis. The sequencing data can be found under SRA Accession: SRR15371771	This study
PE0030	*E. coli* DH10β carries pCRISPR-PE-bacteria, pCDF-GFPplus, pPEgRNA_GFP_TAAin, and pVRb_PEgRNA_312Gdel	This study
PE0031	*E. coli* DH10β carries pCRISPR-PE-bacteria, pCDF-GFPplus, pPEgRNA_GFP_198Tdel, and pVRb_PEgRNA_312Gdel	This study

PE0032	*E. coli* DH10β carries pCRISPR-PE-bacteria, pCDF-GFPplus, pPEgRNA_GFP_T198A, and pVRb_PEgRNA_312Gdel	This study
PE0033	*E. coli* DH10β carries pCRISPR-PE-bacteria, pCDF-GFPplus, pPEgRNA_GFP_combo, and pVRb_PEgRNA_312Gdel	This study
PE0034	*E. coli* DH10β carries pCRISPR-PE-bacteria, pCDF-GFPplus, pPEgRNA_GFP_T196C_T198C, and pVRb_PEgRNA_312Gdel	This study
PE0035	*E. coli* DH10β carries pCRISPR-PE-bacteria, pCDF-GFPplus, and pPEgRNA_GFP_198Tdel. Under induction, it is green (GFP expressed). Whole-genome sequenced for mutation analysis to find clues of editing escaping. Escaper #1. The sequencing data can be found under SRA Accession: SRR15371476	This study
PE0036	*E. coli* DH10β carries pCRISPR-PE-bacteria, pCDF-GFPplus, and pPEgRNA_GFP_198Tdel. Under induction, it is green (GFP expressed). Whole-genome sequenced for mutation analysis to find clues of editing escaping. Escaper #2. The sequencing data can be found under SRA Accession: SRR15371475	This study
PE0037	*E. coli* DH10β carries pCRISPR-PE-bacteria, pCDF-GFPplus, and pPEgRNA_GFP_198Tdel. Under induction, it is green (GFP expressed). Whole-genome sequenced for mutation analysis to find clues of editing escaping. Escaper #3. The sequencing data can be found under SRA Accession: SRR15371474	This study
PE0038	*E. coli* DH10β carries pCRISPR-PE-bacteria, pCDF-GFPplus, and pPEgRNA_GFP_198Tdel. Under induction, it is green (GFP expressed). Whole-genome sequenced for mutation analysis to find clues of editing escaping. Escaper #4. The sequencing data can be found under SRA Accession: SRR15371492	This study
PE0039	*E. coli* DH10β carries pCRISPR-PE-bacteria, pCDF-GFPplus, and pPEgRNA_GFP_198Tdel. Under induction, it is green (GFP expressed). Whole-genome sequenced for mutation analysis to find clues of editing escaping. Escaper #5. The sequencing data can be found under SRA Accession: SRR15371491	This study
PE0040	*E. coli* DH10β carries pCRISPR-PE-bacteria, pCDF-GFPplus, and pPEgRNA_GFP_198Tdel. Under induction, it is green (GFP expressed). Whole-genome sequenced for mutation analysis to find clues of editing escaping. Escaper #6. The sequencing data can be found under SRA Accession: SRR15371490	This study
PE0041	*E. coli* DH10β carries pCRISPR-PE-bacteria, pCDF-GFPplus, and pPEgRNA_GFP_198Tdel. Under induction, it is green (GFP expressed). Whole-genome sequenced for mutation analysis to find clues of editing escaping.	This study

	Escaper #7. The sequencing data can be found under SRA Accession: SRR15371489	
PE0042	*E. coli* DH10β carries pCRISPR-PE-bacteria, pCDF-GFPplus, and pPEgRNA_GFP_198Tdel. Under induction, it is green (GFP expressed). Whole-genome sequenced for mutation analysis to find clues of editing escaping. Escaper #8. The sequencing data can be found under SRA Accession: SRR15371488	This study
PE0043	*E. coli* DH10β carries pCRISPR-PE-bacteria, pCDF-GFPplus, and pPEgRNA_GFP_198Tdel. Under induction, it is green (GFP expressed). Whole-genome sequenced for mutation analysis to find clues of editing escaping. Escaper #9. The sequencing data can be found under SRA Accession: SRR15371487	This study
PE0044	*E. coli* DH10β carries pCRISPR-PE-bacteria, pCDF-GFPplus, and pPEgRNA_GFP_198Tdel. Under induction, it is green (GFP expressed). Whole-genome sequenced for mutation analysis to find clues of editing escaping. Escaper #10. The sequencing data can be found under SRA Accession: SRR15371486	This study
PE0045	*E. coli* DH10β carries pCDF-GFPplus. Induced. Whole-genome sequenced as a control. The sequencing data can be found under SRA Accession: SRR15371485	This study
PE0046	*E. coli* DH10β carries pCRISPR-PE-bacteria. Induced. Whole-genome sequenced as a control. The sequencing data can be found under SRA Accession: SRR15371484	This study
PE0047	*E. coli* DH10β carries pCDF-GFPplus and pCRISPR-PE-bacteria. Induced. Whole-genome sequenced as a control. The sequencing data can be found under SRA Accession: SRR15371483	This study
PE0048	*E. coli* DH10β carries pCDF-GFPplus, pCRISPR-PE-bacteria, and a pPEgRNA without the 26-bp 3 prime extension. Induced, the clone is green. Whole-genome sequenced as a control. The sequencing data can be found under SRA Accession: SRR15371481	This study

Supplementary Table 2. Primers used in this study

Names	Sequence (5' to 3')	Purpose
PEgRNA_GFP_F	gtcctaggtataatactagtCTTGTCACTACTCTGACCTAgttttagagctagaaatagc	For PCR amplification of desired PEgRNA functional cassettes. Sequences in lower case are overhangs for Gibson assembly.
PEgRNA_GFP_TAAin_R	gggcccaagcttcaaaaaaaTCACTACTCTGACCTATTAAGGTGTTCAAgcaccgactcggtgccactt	
PEgRNA_GFP_TAAin_T4_R	gggcccaagcttcaaaaaaaTCACTACTCTGACCTATTAAgcaccgactcggtgccactt	
PEgRNA_GFP_TAAin_T5_R	gggcccaagcttcaaaaaaaTCACTACTCTGACCTATTAAGgcaccgactcggtgccactt	
PEgRNA_GFP_TAAin_T8_R	gggcccaagcttcaaaaaaaTCACTACTCTGACCTATTAAGGTGgcaccgactcggtgccactt	
PEgRNA_GFP_TAAin_T20_R	gggcccaagcttcaaaaaaaTCACTACTCTGACCTATTAAGGTGTTCAATGCTTTTgcaccgactcggtgccactt	
PEgRNA_GFP_TAAin_PBS5_R	gggcccaagcttcaaaaaaCTGACCTATTAAGGTGTTCAAgcaccgactcggtgccactt	
PEgRNA_GFP_TAAin_PBS8_R	gggcccaagcttcaaaaaaaACTCTGACCTATTAAGGTGTTCAAgcaccgactcggtgccactt	
PEgRNA_GFP_TAAin_PBS17_R	gggcccaagcttcaaaaaaaACTCTGACCTATTAAGGTGTTCAAgcaccgactcggtgccactt	
PEgRNA_GFP_T198A_R	gggcccaagcttcaaaaaaaTCACTACTCTGACCTAAGGTGTTCAAgcaccgactcggtgccactt	
PEgRNA_GFP_T196C_T198C_R	gggcccaagcttcaaaaaaaTCACTACTCTGACCCACGGTGTTCAAgcaccgactcggtgccactt	

PEgRNA_GFP_198Tdel_R	gggcccaagcttcaaaaaaaTCACTACTCTGACCTAGGTGTTCAATgcaccgactcggtgccactt	
PEgRNA_GFP_combo_R	gggcccaagcttcaaaaaaaTCACTACTCTGACCTAATAAGTGTTCAAgcaccgactcggtgccactt	
PEgRNA_GFP_12in_R	gggcccaagcttcaaaaaaaTCACTACTCTGACCTATGTAATCTGTACAGGTGTTCAAgcaccgactcggtgccactt	
PEgRNA_GFP_18in_R	gggcccaagcttcaaaaaaaTCACTACTCTGACCTATTAATACGACTCACTATAGGGTGTTCAAgcaccgactcggtgccactt	
PEgRNA_GFP_33in_R	gggcccaagcttcaaaaaaaTCACTACTCTGACCTATTGTAGAATCAGCCCACGAAACCGAGCGGGCGAGGGTGTTCAAgcaccgactcggtgccactt	
PEgRNA_GFP_10del_R	gggcccaagcttcaaaaaaaTCACTACTCTGACATATCTTTCAAAGgcaccgactcggtgccactt	
PEgRNA_GFP_23del_R	gggcccaagcttcaaaaaaaTCACTACTCTGACATATCTTTCAAAGgcaccgactcggtgccactt	
PEgRNA_GFP_36del_R	gggcccaagcttcaaaaaaaTCACTACTCTGACATATCTTTCAAAGgcaccgactcggtgccactt	
PEgRNA_GFP_49del_R	gggcccaagcttcaaaaaaaTCACTACTCTGACATATCTTTCAAAGgcaccgactcggtgccactt	
PEgRNA_GFP_97del_R	gggcccaagcttcaaaaaaaTCACTACTCTGACATATCTTTCAAAGgcaccgactcggtgccactt	
PEgRNA_GFP_312Gdel_F	gtcctaggtataatactagtCTATATCTTTCAAAGATGACgttttagagctagaaatagc	For PCR amplification of GFP 312G deletion PEgRNA for Gibson assembly.
PEgRNA_GFP_312Gdel_R	gggcccaagcttcaaaaaaaATCTTTCAAAGATGACGGAACTACAAGCACCGACTCGGTGCCACTT	
pVRb_PEgRNA_backbone_F	TTTTTTTGAAGCTTGGGCCC	For PCR amplification of plasmid

pVRb_PEgRNA_backbone_R	ACTAGTATTATACCTAGGACTGAGC	backbone for Gibson assembly of PEgRNA.
pPEgRNA_lacZ_TAGin_F	gtcctaggtataatactagtAATCCCGAATCTCTATCGTGgttttagagctagaaatagc	For PCR amplification of desired functional PEgRNA_lacZ_TAGincassettes. Sequences in lower case are overhangs for Gibson assembly.
pPEgRNA_lacZ_TAGin_R	gggcccaagcttcaaaaaaCCGAATCTCTATCGTGCGTAGGTGGTTGA gcaccgactcggtgccactt	
pPEgRNA_lacZ_CGdel_F	gtcctaggtataatactagtTATGCAGCAACGAGACGTCAgttttagagctagaaatagc	For PCR amplification of desired functional PEgRNA_lacZ_CGde cassettes. Sequences in lower case are overhangs for Gibson assembly.
pPEgRNA_lacZ_CGdel_R	gggcccaagcttcaaaaaaGCAGCAACGAGACGTCAGAAAATGCCGCTCATgcaccgactcggtgccactt	
pPEgRNA_lacZ_GTtoTAsub_F	gtcctaggtataatactagtGCGAGTTGCGTGACTACCTAgttttagagctagaaatagc	For PCR amplification of desired functional PEgRNA_lacZ_GTtoTAsub cassettes. Sequences in lower case are overhangs for Gibson assembly.
pPEgRNA_lacZ_GTtoTAsub_R	gggcccaagcttcaaaaaaAGTTGCGTGACTACTAACGGGTAACAGTgcaccgactcggtgccactt	
pPEgRNA_galK_TAAin_F	gtcctaggtataatactagtGACAGCCACACCTTTGGGCAgttttagagctagaaatagc	For PCR amplification of desired functional PEgRNA_galK_TAAin cassettes. Sequences in lower case are overhangs for Gibson assembly.
pPEgRNA_galK_TAAin_R	gggcccaagcttcaaaaaaGCCACACCTTTGGGCATTTAGGAAACTGCgcaccgactcggtgccactt	
lacZ_check_F	gatgaaagctggctacagga	For PCR amplification of the targeted

lacZ_check_F	tgacggttaacgcctcgaat	region in *lacZ* gene. The forward primer is also used for Sanger sequencing.
galK_check_F	caatgggctaactacgttcg	For PCR amplification of the targeted region in *galK* gene. The forward primer is also used for Sanger sequencing.
galK_check_F	gtcgccaatcacagctttga	
pnsgRNA_GFP_TAAin_F	AAAGCATTGAACACCttaATGTTTTAGAGCTAGAAATAGC	For PCR amplification of the nsgRNA_GFP_TAAin cassette.
pnsgRNA_GFP_TAAin_R	ATtaaGGTGTTCAATGCTTTactagtattatacctaggac	
pnsgRNA_lacZ_TAGin_F	CctaCGCACGATAGAGATTCGTTTTAGAGCTAGAAATAGC	For PCR amplification of the nsgRNA_lacZ_TAGin cassette.
pnsgRNA_lacZ_TAGin_R	GAATCTCTATCGTGCGtagGactagtattatacctaggac	
pnsgRNA_lacZ_CGdel_F	CTGGAGTGACGGCAGTTATCGTTTTAGAGCTAGAAATAGC	For PCR amplification of the nsgRNA_lacZ_CGdel cassette.
pnsgRNA_lacZ_CGdel_R	GATAACTGCCGTCACTCCAGactagtattatacctaggac	
pnsgRNA_lacZ_GTtoTAsub_F	CATTAAAGCGAGTGGCAACAGTTTTAGAGCTAGAAATAGC	For PCR amplification of the nsgRNA_lacZ_GTtoTAsub cassette.
pnsgRNA_lacZ_GTtoTAsub_R	TGTTGCCACTCGCTTTAATGactagtattatacctaggac	

pVRb_backbone_F	CTGTTGTTTGTCGGTGAACG	For PCR amplification of the pVRb backbone from pVRb_20_992.
pVRb_backbone_R	AAGGGCCTCGTGATACGCCT	
sgRNA_cassette_F	AGGCGTATCACGAGGCCCTTgaattctaaagatctttgac	For PCR amplification of the sgRNA cassette from pPEgRNA.
sgRNA_cassette_R	CGTTCACCGACAAACAACAGataaaacgaaaggcccagtc	
nsgRNA_seq	GCAATTCCGACGTCTAAG	For validation of the Gibson assembly of pnsgRNA.<br>Also for validation of PEgRNA cloning of pVRb_PEgRNA.
PEgRNA_backbone_F	TTTTTTTGAAGCTTGGGCCC	For PCR amplification of the pPEgRNA backbone
PEgRNA_backbone_R	ACTAGTATTATACCTAGGAC	
PEgRNA_F	GTCCTAGGTATAATACTAGTGTTTTAGAGCTAGAAATAGCA	For removal of the 20 bp spacer from the plasmid pgRNA-bacteria.
PEgRNA_R	ACTAGTATTATACCTAGGACTGAGCTAGCT	
PEgRNA_Seq	AATAGGCGTATCACGAGGCA	For Sanger sequencing of correctly assembled PEgRNA plasmids.
pCDF-GFP_Seq	GAAATACTAGATGAGCAAAGGAGAAG	For screening of edited events using Sanger sequencing.

pCDF-1b_F	GTATATCTCCTTATTAAAGT	For PCR amplification of the pCDF-1b backbone.
pCDF-1b_R	ATTAACCTAGGCTGCTGCCA	
GFPplus_F	ACTTTAATAAGGAGATATACTTTACGGCTAGCTCAGTCCT	For PCR amplification of the J23106-GFPplus-T0 cassette.
GFPplus_R	TGGCAGCAGCCTAGGTTAATCGAACCGAACAGGCTTATGT	
GFPplus_check_F	ATGCGACTCCTGCATTAGGA	For screening of correctly assembled pCDF-GFPplus using PCR. The GFPplus_check_F is also used for Sanger sequencing validation.
GFPplus_check_R	ACTAGTCGCCAGGGTTTTCC	
A10D_F	TAGGCTTAGATATCGGCACAAA	For site-directed mutating of 10A to 10D with the dCas9 in the pdCas9-bacteria.
A10D_R	TTGAGTATTTCTTATCCATATG	
D10_Seq	GCGAGTTTACGGGTTGTTA	For screening of correct mutations of 10A to 10D using Sanger sequencing.
pCas9n_F	TAACTCGAGTAAGGATCTCC	For PCR amplification of the pCas9n(H840A) backbone, the stop codon of Cas9n is removed.
pCas9n_R	GTCACCTCCTAGCTGACTCA	

EcMMLV2_F	TGAGTCAGCTAGGAGGTGAC	For PCR amplification of the *E. coli* codon optimized linker-M-MLV2 fragment.
EcMMLV2_R	GGAGATCCTTACTCGAGTTA	
EcMMLV2_check_R	AACAAACAGCTCGAACGGCT	Together with EcMMLV2_F, this primer set is used for PCR screening of *E. coli* codon optimized linker-M-MLV2 fragment insertion.
EcMMLV2_seq	AACTGGATTGCCAACAGGGT	Together with EcMMLV2_F, these three primers are used to confirm the insertion of *E. coli* codon optimized linker-M-MLV2 fragment by Sanger sequencing.
dblTerm_R_seq	GAAGGTGAGCCAGTGTGACT	

Supplementary Table 3. Important sequences involved in this study

Name	Sequence	Description
J23106-GFPplus-T0 cassette	TTTACGGCTAGCTCAGTCCT<br>AGGTATAGTGCTAGCTACTA<br>GAGAAAGAGGAGAAATACTA<br>GATGAGCAAAGGAGAAGAA<br>CTTTTCACTGGAGTTGTCCC<br>AATTCTTGTTGAATTAGATG<br>GTGATGTTAATGGGCACAAA<br>TTTTCTGTCAGTGGAGAGGG<br>TGAAGGTGATGCTACATACG<br>GAAAACTCACCCTTAAATTT<br>ATTTGCACTACTGGAAAACT<br>ACCTGTTCCATGGCCAACAC<br>TTGTCACTACTCTGACCTAT<br>GGTGTTCAATGCTTTTCCCG<br>TTATCCGGATCACATGAAAC<br>GGCATGACTTTTTCAAGAGT<br>GCCATGCCCGAAGGTTATGT<br>ACAGGAACGCACTATATCTT<br>TCAAAGATGACGGGAACTAC<br>AAGACGCGTGCTGAAGTCA<br>AGTTTGAAGGTGATACCCTT<br>GTTAATCGTATCGAGTTAAA<br>GGGTATTGATTTTAAAGAAG<br>ATGGAAACATTCTCGGACAC<br>AAACTAGAGTACAACTATAA<br>CTCACACAATGTATACATCA<br>CGGCAGACAAACAAAAGAAT<br>GGAATCAAAGCTAACTTCAA<br>AATTCGCCACAACATTGAAG<br>ATGGTTCCGTTCAACTAGCA<br>GACCATTATCAACAAAATAC<br>TCCAATTGGCGATGGCCCT<br>GTCCTTTTACCAGACAACCA<br>TTACCTGTCGACACAATCTG<br>CCCTTTCGAAAGATCCCAAC<br>GAAAAGCGTGACCACATGG<br>TCCTTCTTGAGTTTGTAACT<br>GCTGCTGGGATTACACATG<br>GCATGGATGAGCTCTACAAA<br>TGAAGCGCATACCTGCAGG<br>CATGCAAGCTTGCGGCCGC<br>GTCGTGACTGGGAAAACCC<br>TGGCGACTAGTCTTGGACTC<br>CTGTTGATAGATCCAGTAAT<br>GACCTCAGAACTCCATCTGG<br>ATTTGTTCAGAACGCTCGGT<br>TGCCGCCGGGCGTTTTTTAT<br>TGGTGAGAATCCAGGGGTC	A fast folding GFP variant GFP+ encoding gene (in blue), controlled by a constitutive promoter J23106 (in green) and ended with a T0 terminator (in orange).

	CCCAATAATTACGATTTAAAT<br>TTGACATAAGCCTGTTCGGT<br>TCG	
E. coli codon optimized linker-M-MLV2 fragment	*TGAGTCAGCTAGGAGGTGA*<br>*C*AGCGGCGGCAGCAGCGG<br>CGGCAGCAGCGGCAGCGAA<br>ACCCCGGGCACCAGCGAAA<br>GCGCGACCCCGGAAAGCAG<br>CGGCGGCAGCAGCGGCGG<br>TAGCAGCACCCTGAACATCG<br>AGGACGAGTATCGTCTGCAT<br>GAGACCAGCAAGGAGCCGG<br>ATGTTAGCCTGGGTAGCACC<br>TGGCTGAGCGACTTTCCGC<br>AGGCGTGGGCGGAAACCGG<br>CGGCATGGGTCTGGCGGTT<br>CGCCAGGCGCCGCTGATCA<br>TTCCGCTGAAGGCGACCAG<br>CACCCCGGTTAGCATCAAG<br>CAGTATCCGATGAGCCAGG<br>AAGCGCGTCTGGGTATTAAG<br>CCGCACATTCAACGTCTGCT<br>GGACCAGGGTATTCTGGTG<br>CCGTGCCAGAGCCCGTGGA<br>ATACCCCGCTGCTGCCGGT<br>GAAGAAACCGGGTACCAAT<br>GATTACCGTCCGGTGCAAG<br>ACCTGCGTGAGGTTAACAAG<br>CGCGTTGAAGATATTCATCC<br>GACCGTTCCGAACCCGTAC<br>AACCTGCTGAGCGGTCTGC<br>CGCCGAGCCACCAGTGGTA<br>TACCGTGCTGGATCTGAAG<br>GACGCGTTTTTCTGCCTGCG<br>TCTGCACCCGACCAGCCAA<br>CCGCTGTTCGCGTTTGAATG<br>GCGTGACCCGGAAATGGGT<br>ATCAGCGGCCAACTGACCT<br>GGACCCGTCTGCCGCAGGG<br>CTTTAAAAACAGCCCGACCC<br>TGTTCAACGAGGCGCTGCA<br>CCGTGATCTGGCGGACTTC<br>CGTATCCAACACCCGGATCT<br>GATCCTGCTGCAGTACGTG<br>GACGATCTGCTGCTGGCGG<br>CGACCAGCGAACTGGATTG<br>CCAACAGGGTACCCGTGCG<br>CTGCTGCAGACCCTGGGTA<br>ACCTGGGTTACCGTGCGAG<br>CGCGAAAAAGGCGCAAATTT<br>GCCAGAAGCAAGTGAAGTAT	A 33-amino acid linker (in red) fused with the M-MLV2 encoding sequence (in black). 20 bp overhangs for Gibson assembly into pCas9n(H840A) is shown in gray italic.

	CTGGGCTACCTGCTGAAGG<br>AAGGTCAACGCTGGCTGAC<br>CGAGGCGCGTAAGGAAACC<br>GTTATGGGTCAGCCGACCC<br>CGAAGACCCCGCGCCAACT<br>GCGTGAGTTCCTGGGTAAA<br>GCGGGTTTTTGCCGTCTGTT<br>TATCCCGGGTTTCGCGGAAA<br>TGGCGGCGCCGCTGTACCC<br>GCTGACCAAACCGGGTACC<br>CTGTTTAACTGGGGTCCGGA<br>CCAGCAGAAAGCGTACCAA<br>GAGATCAAACAGGCGCTGC<br>TGACCGCGCCGGCGCTGGG<br>TCTGCCGGACCTGACCAAG<br>CCGTTCGAGCTGTTTGTTGA<br>TGAAAAGCAGGGTTATGCGA<br>AAGGCGTTCTGACCCAGAAA<br>CTGGGTCCGTGGCGCCGTC<br>CGGTTGCGTACCTGAGCAA<br>GAAACTGGATCCGGTTGCG<br>GCGGGCTGGCCGCCGTGCC<br>TGCGTATGGTTGCGGCGAT<br>CGCGGTTCTGACCAAAGAC<br>GCGGGCAAGCTGACCATGG<br>GTCAACCGCTGGTGATTCTG<br>GCGCCGCATGCGGTTGAAG<br>CGCTGGTTAAGCAGCCGCC<br>GGACCGTTGGCTGAGCAAC<br>GCGCGTATGACCCACTATCA<br>AGCGCTGCTGCTGGATACC<br>GACCGTGTTCAGTTCGGTCC<br>GGTGGTTGCGCTGAACCCG<br>GCGACCCTGCTGCCGCTGC<br>CGGAGGAAGGTCTGCAGCA<br>TAACTGCCTGGACATTCTGG<br>CGGAGGCGCACGGTACCCG<br>TCCGGATCTGACCGACCAG<br>CCGCTGCCGGACGCGGATC<br>ACACCTGGTATACCGACGG<br>CAGCAGCCTGCTGCAAGAA<br>GGCCAGCGTAAGGCGGGTG<br>CGGCGGTTACCACCGAGAC<br>CGAAGTTATCTGGGCGAAA<br>GCGCTGCCGGCGGGTACCA<br>GCGCGCAGCGTGCGGAGCT<br>GATTGCGCTGACCCAAGCG<br>CTGAAAATGGCGGAGGGCA<br>AAAAGCTGAATGTTTATACC<br>GATAGCCGTTACGCGTTTGC<br>GACCGCGCACATCCATGGT	

	GAAATCTACCGTCGTCGTGGTTGGCTGACCAGCGAAGGCAAAGAAATCAAAAATAAGGACGAGATTCTGGCGCTGCTGAAAGCGCTGTTCCTGCCGAAACGTCTGAGCATCATTCACTGCCCGGGTCACCAGAAAGGTCACAGCGCGGAGGCGCGTGGTAATCGCATGGCGGATCAAGCGGCGCGTAAAGCGGCGATTACCGAAACCCCGGATACCAGCACCCTGCTGATTGAAAATAGCAGCCCGTAA*TAACTCGAGTAAGGATCTCC*	
An example (TAAin) of functional PEgRNA cassette	ttgacagctagctcagtcctaggtataatactagtCTTGTCACTACTCTGACCTAGTTTTAGAGCTAGAAATAGCAAGTTAAAATAAGGCTAGTCCGTTATCAACTTGAAAAAGTGGCACCGAGTCGGTGCTTGAACACC***TTA***ATAGGTCAGAGTAGTGAtttttt	Green: J23119 promoter<br>Red: 20-nt spacer targeting GFPplus coding sequence<br>Black: 76-nt gRNA scaffold<br>Blue: RTT of the 3 prime extension, the target TAA is shown in bold, italic<br>Orange: PBS of the 3 prime extension

Supplementary Table 4. Spacers and 3 prime extensions used in this study

PEgRNA	Space (5'-3')	3 prime extension (5'-3')	RTT length (nt)	PBS length (nt)
PEgRNA_GFP_TAAin_T4	CTTGTCACTACTCTGACCTA	TTAATAGGTCAGAGTAGTGA	7	13
PEgRNA_GFP_TAAin_T5	CTTGTCACTACTCTGACCTA	CTTAATAGGTCAGAGTAGTGA	8	13
PEgRNA_GFP_TAAin_T8	CTTGTCACTACTCTGACCTA	CACCTTAATAGGTCAGAGTAGTGA	11	13
PEgRNA_GFP_TAAin	CTTGTCACTACTCTGACCTA	TTGAACACCTTAATAGGTCAGAGTAGTGA	16	13
PEgRNA_GFP_TAAin_T20	CTTGTCACTACTCTGACCTA	AAAAGCATTGAACACCTTAATAGGTCAGAGTAGTGA	23	13
PEgRNA_GFP_TAAin_PBS5	CTTGTCACTACTCTGACCTA	TTGAACACCTTAATAGGTCAG	16	5
PEgRNA_GFP_TAAin_PBS8	CTTGTCACTACTCTGACCTA	TTGAACACCTTAATAGGTCAGAGT	16	8
PEgRNA_GFP_TAAin_PBS17	CTTGTCACTACTCTGACCTA	TTGAACACCTTAATAGGTCAGAGTAGTGACAAG	16	17
PEgRNA_GFP_T198A	CTTGTCACTACTCTGACCTA	TTGAACACCTTAGGTCAGAGTAGTGA	13	13
PEgRNA_GFP_T196C_T198C	CTTGTCACTACTCTGACCTA	TTGAACACCGTGGGTCAGAGTAGTGA	13	13
PEgRNA_GFP_198Tdel	CTTGTCACTACTCTGACCTA	ATTGAACACCTAGGTCAGAGTAGTGA	13	13
PEgRNA_GFP_combo	CTTGTCACTACTCTGACCTA	TTGAACACTTATTAGGTCAGAGTAGTGA	15	13

PEgRNA_GFP_12in	CTTGTCACTACTCTGACCTA	TTGAACACCTGTACAGATTACATAGGTCAGAGTAGTGA	25	13
PEgRNA_GFP_18in	CTTGTCACTACTCTGACCTA	TTGAACACCCTATAGTGAGTCGTATTAATAGGTCAGAGTAGTGA	31	13
PEgRNA_GFP_33in	CTTGTCACTACTCTGACCTA	TTGAACACCCTCGCCCGCTCGGTTTCGTGGGCTGATTCTACAATAGGTCAGAGTAGTGA	46	13
PEgRNA_GFP_10del	CTTGTCACTACTCTGACCTA	GGGAAAAGCATTGGTCAGAGTAGTGA	13	13
PEgRNA_GFP_23del	CTTGTCACTACTCTGACCTA	TGATCCGGATAACGTCAGAGTAGTGA	13	13
PEgRNA_GFP_36del	CTTGTCACTACTCTGACCTA	ATGCCGTTTCATGGTCAGAGTAGTGA	13	13
PEgRNA_GFP_49del	CTTGTCACTACTCTGACCTA	TCTTGAAAAAGTCGTCAGAGTAGTGA	13	13
PEgRNA_GFP_97del	CTTGTCACTACTCTGACCTA	CTTTGAAAGATATGTCAGAGTAGTGA	13	13
PEgRNA_lacZ_TAGin	AATCCCGAATCTCTATCGTG	TCAACCACCTACGCACGATAGAGATTCGG	16	13
PEgRNA_lacZ_CGdel	TATGCAGCAACGAGACGTCA	ATGAGCGGCATTTTCTGACGTCTCGTTGCTGC	18	14
PEgRNA_lacZ_GTtoTAsub	GCGAGTTGCGTGACTACCTA	ACTGTTACCCGTTAGTAGTCACGCAACT	14	14
PEgRNA_galK_TAAin	GACAGCCACACCTTTGGGCA	GCAGTTTCCTAAATGCCCAAAGGTGTGGC	16	13
PEgRNA_GFP_312Gdel	CTATATCTTTCAAAGATGAC	TTGTAGTTCCGTCATCTTTGAAAGAT	13	13

nsgRNA	PE3 or PE3b[1,10]	Space (5’-3’)		
nsgRNA_GFP_TAAin	PE3b	AAAGCATTGAACACCttaAT		
nsgRNA_lacZ_TAGin	PE3b	CctaCGCACGATAGAGATTC		
nsgRNA_lacZ_CGdel	PE3	CTGGAGTGACGGCAGTTATC		
nsgRNA_lacZ_GTtoTAsub	PE3	CATTAAAGCGAGTGGCAACA		

Supplementary Table 5. A CFU assay of *E. coli* strains transformed with different plasmids

**Plasmid**	**LB Plate** (200 ng/ml of ATc, 100 ug/ml of Spec and Amp, and 25 ug/ml of Chl were used)	**CFU (Mean ± SD) n=3**	**Induction/non-induction**
pCDF-GFP	Spec	12800000 ± 1552417.47	
pCDF-GFP	Spec + ATc	10333333.3 ± 960902.35	(82.1 ± 17.3)%
pCDF-GFP + pCRISPR-PE	Spec + Chl	21810 ± 1174.61	
pCDF-GFP + pCRISPR-PE	Spec + Chl + ATc	17550 ± 2255.39	(80.8 ± 13.4)%
pCDF-CDF + pCRISPR-PE + pPEgRNA-GFP-del	Spec + Chl + Amp	1830 ± 542.49	
pCDF-GFP + pCRISPR-PE + pPEgRNA-GFP-del	Spec + Chl + Amp + ATc	1110 ± 274.95	(67.9 ± 36.6)%
pCDF-GFP + pCRISPR-PE + pPEgRNA	Spec + Chl + Amp	6510 ± 1188.32	
pCDF-GFP + pCRISPR-PE + pPEgRNA	Spec + Chl + Amp + ATc	1470 ± 274.95	(23.4 ± 7.1)%

White background: not induced; grey background: induced with ATc.

Supplementary Table 6. The max. doubling time of strains with and without inducer, data are mean of the 12 repeats of each sample

	Induced			Uninduced		
	**pCDF-GFP**	**pCDF-GFP+ pCRISPR-PE+ pPEgRNA**	**pCDF-GFP+ pCRISPR-PE+ pPEgRNA-GFPdel**	**pCDF-GFP**	**pCDF-GFP+ pCRISPR-PE+ pPEgRNA**	**pCDF-GFP+ pCRISPR-PE+ pPEgRNA-GFPdel**
**Slope [h^-1]**	0.6948	0.5285	0.5288	0.6712	0.6139	0.5867
**R squared**	0.9801	0.9816	0.9836	0.9792	0.9813	0.9875
**max. doubling time [h]**	0.9976	1.3115	1.3108	1.0327	1.1291	1.1814

Supplementary Table 7. Whole-genome sequencing-based analysis of escapers from the CRISPR-Prime Editing

Strain	Recorded mutations[a]				SRA Accession
	On-plasmids			On-chromosome	
	pCDF-GFPplus	pPEgRNA_GFP_198Tdel	pCRISPR-PE-bacteria		
PE0035	0	0	0	0	SRR15371476
PE0036	0	2 (Pos. 126. -26 bp, the 3 prime extension was missing)	0	0	SRR15371475
PE0037	0	2 (Pos. 126. -26 bp, the 3 prime extension was missing)	0	0	SRR15371474
PE0038	0	0	0	0	SRR15371492
PE0039	0	0	0	0	SRR15371491
PE0040	0	0	0	0	SRR15371490
PE0041	0	0	0	0	SRR15371489
PE0042	0	0	0	0	SRR15371488
PE0043	0	1 (Pos. 126. -26 bp, the 3 prime extension was missing)	0	0	SRR15371487
PE0044	0	0	0	0	SRR15371486
PE0045	0	-	-	0	SRR15371485
PE0046	-	-	0	0	SRR15371484
PE0047	0	-	0	0	SRR15371483
PE0048	0	1 (Pos. 126. -26 bp, no 3 prime extension was cloned)	0	0	SRR15371481

[a]: the shared mutations were listed here.

One shared mutation in pCRISPR-PE-bacteria is Pos. 8,584. A43A (GCC→GCA);

One shared mutation in pPEgRNA_GFP_198Tdel is Pos. 1,146. G→A, intergenic, 6XHis;

One shared mutation in the chromosome is Pos. 4,272,972. +T, intergenic.

Supplementary Table 8. Overview of generated illumina datasets

Parental strain	NCBI sample name	SRA Accession	Names in Table S7 and S1
K-12 substr. DH10B	YT_gp1	SRR15371494	PE0005
K-12 substr. DH10B	YT_gp2	SRR15371493	PE0016
K-12 substr. DH10B	YT_gp3	SRR15371482	PE0013
K-12 substr. DH10B	YT_gp4	SRR15371480	PE0014
K-12 substr. DH10B	YT_gp5	SRR15371479	PE0015
K-12 substr. DH10B	YT_gp6	SRR15371478	PE0017
K12 substr. MG1655	YT_g7	SRR15371774	PE0026
K12 substr. MG1655	YT_g8	SRR15371773	PE0027
K12 substr. MG1655	YT_g9	SRR15371772	PE0028
K12 substr. MG1655	YT_g10	SRR15371771	PE0029
K-12 substr. DH10B	YT_g11	SRR15371477	Escherichia coli DH10β (DH10B)
K12 substr. MG1655	YT_g12	SRR15371770	Escherichia coli MG1655
K-12 substr. DH10B	cwi15	SRR15371476	PE0035
K-12 substr. DH10B	cwi16	SRR15371475	PE0036
K-12 substr. DH10B	cwi17	SRR15371474	PE0037
K-12 substr. DH10B	cwi18	SRR15371492	PE0038
K-12 substr. DH10B	cwi19	SRR15371491	PE0039
K-12 substr. DH10B	cwi20	SRR15371490	PE0040
K-12 substr. DH10B	cwi21	SRR15371489	PE0041
K-12 substr. DH10B	cwi22	SRR15371488	PE0042
K-12 substr. DH10B	cwi23	SRR15371487	PE0043
K-12 substr. DH10B	cwi24	SRR15371486	PE0044
K-12 substr. DH10B	cwi25	SRR15371485	PE0045
K-12 substr. DH10B	cwi26	SRR15371484	PE0046
K-12 substr. DH10B	cwi27	SRR15371483	PE0047
K-12 substr. DH10B	cwi28	SRR15371481	PE0048

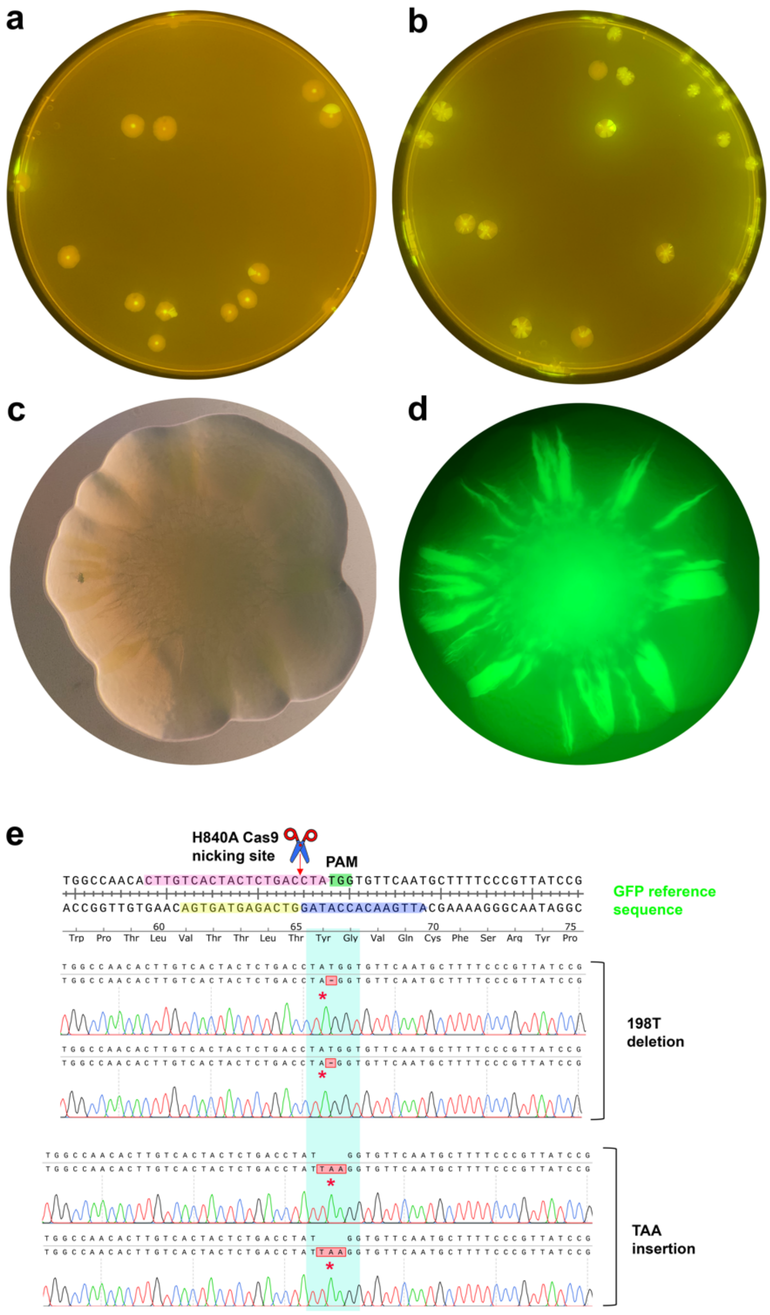

Supplementary Figure 1. Editing accumulates over time of using CRISPR-Prime Editing system in *E. coli*.

- **a.** A five-day old induction (200 ng/mL ATc) plate of GFP 1-bp deletion under a Blue-Light Transilluminator (Safe Imager 2.0, Thermo Fisher Scientific, US).
- **b.** A five-day old induction (200 ng/mL ATc) plate of GFP 3-bp insertion under a Blue-Light Transilluminator (Safe Imager 2.0, Thermo Fisher Scientific, US).

c. The 40x optical view of a single colony from **b.** under a Leica DM4000 B Fluorescence Microscope (Leica Microsystems, Germany)

d. The 40x GFP fluorescent view of the same colony as **c.** using the same microscope.

e. Two of each outgrown colony from **a.** and **b.** were Sanger-sequenced. The in-figure legend is the same as Fig. 2a.

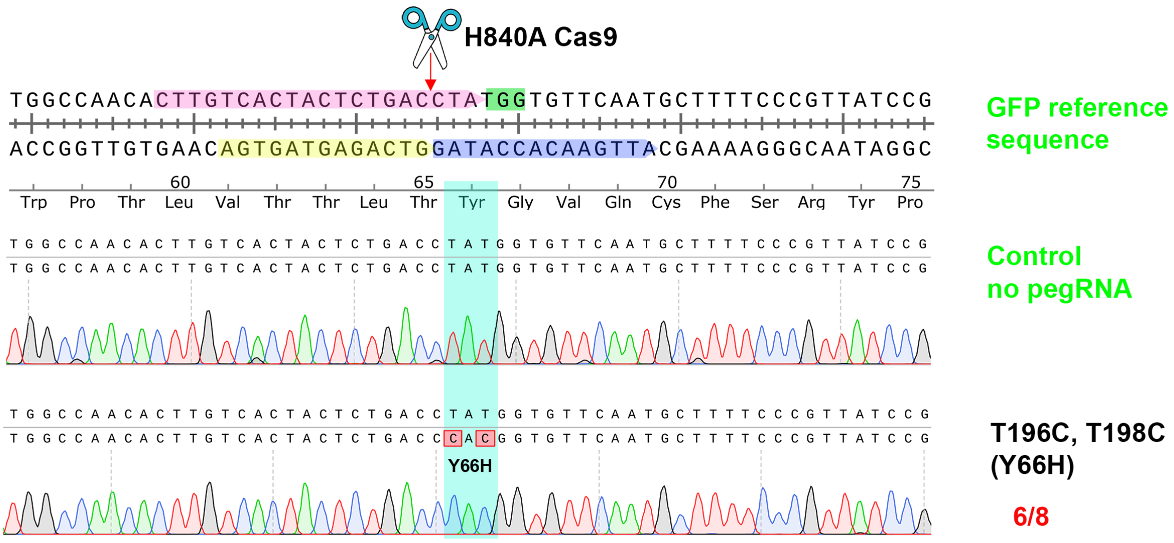

Supplementary Figure 2. A DNA editing of double substitutions by CRISPR-Prime Editing.

Eight randomly picked colonies of each designed DNA engineering were Sanger sequenced and traces were aligned to the targeted locus of GFP coding sequence. The correctly edited colony numbers and the total sequenced numbers were shown in red. The in-figure legend is the same as Fig. 2a.

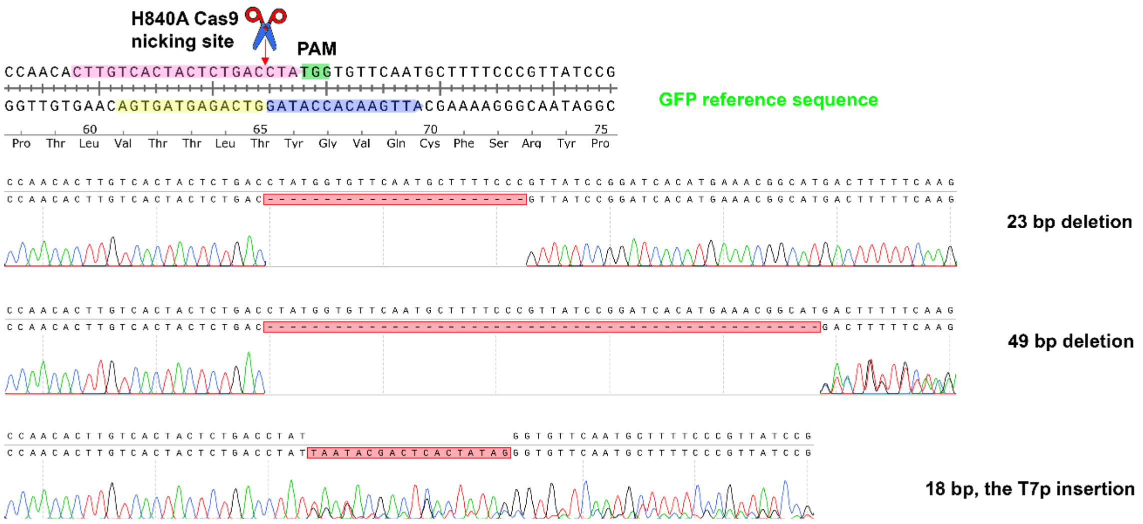

Supplementary Figure 3. Long DNA fragments deletion and insertions using CRISPR-Prime Editing in *E. coli*.

As examples, Sanger sequencing traces of a 23-bp deletion, a 49-bp deletion, and a 18-bp (mini-T7 promoter) insertion traces were aligned to the targeted locus of GFP coding sequence. The in-figure legend is the same as Fig. 2a.

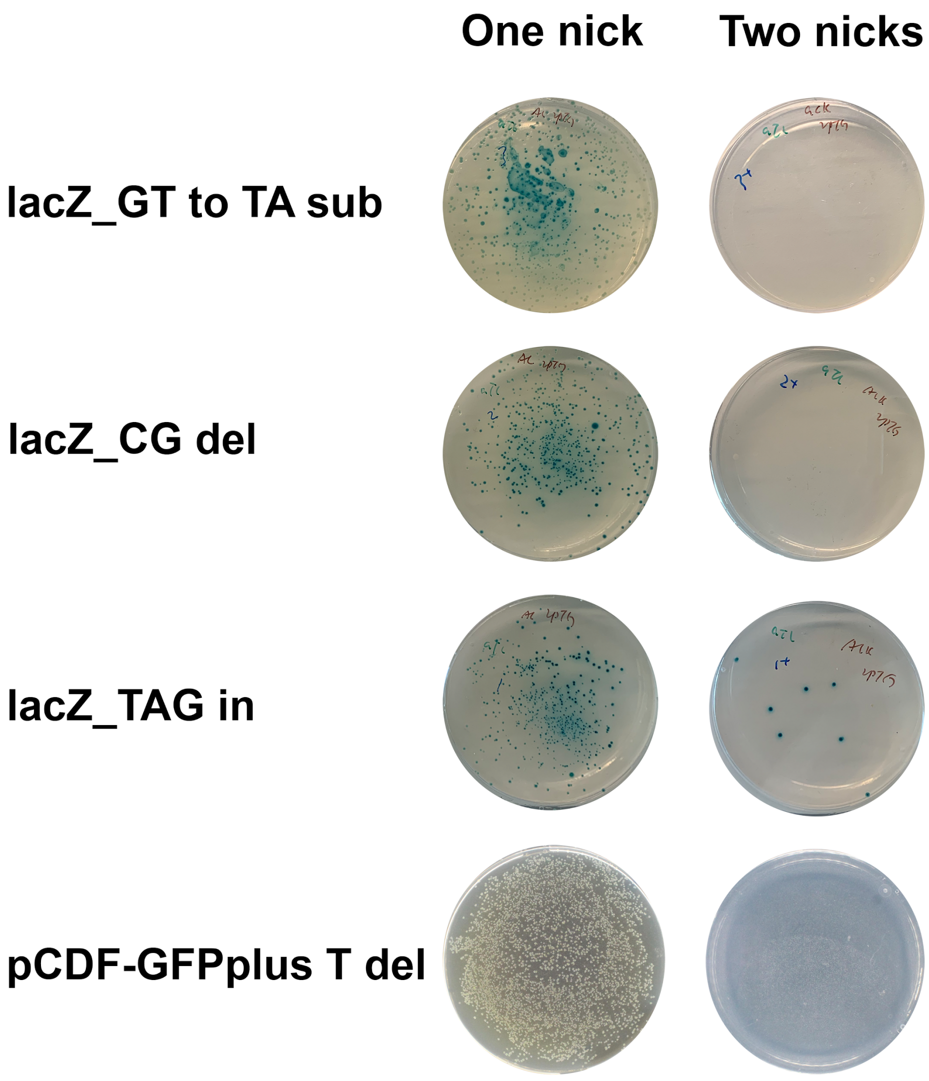

Supplementary Figure 4. A second nick compromises the application of CRISPR-Prime Editing in *E. coli.*

Plates showed the colony formation of transformants. For the one nick panel, 50 μl of transformation culture was plated onto appropriate antibiotics supplemented LB plates, while for the two nicks panel, 400 μl of transformation culture was plated. Photos were taken by a Doc-It imaging station after 24h incubation at 37 °C.

a

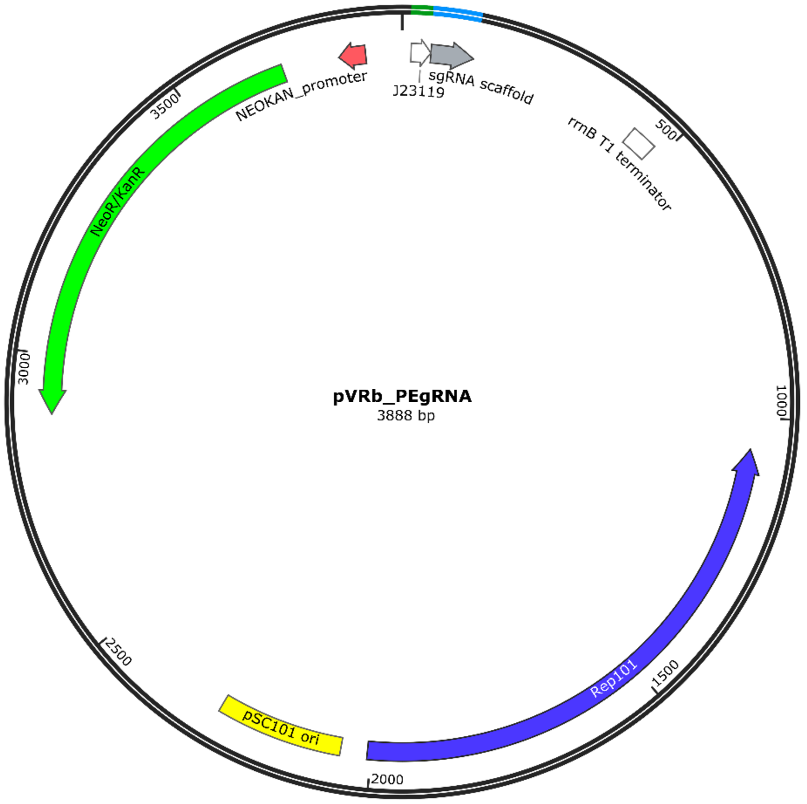

b

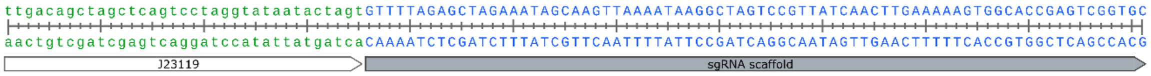

Supplementary Figure 5. The plasmid map of pVRb_PEgRNA.

a. The plasmid map of the pVRb_PEgRNA plasmid, which is pSC101 ori, kanamycin resistant, and with a J23119 driven sgRNA scaffold.
b. The DNA sequence of the J23119-sgRNA scaffold is displayed. The J23119 promoter sequence is in green, and the sgRNA scaffold is in blue.

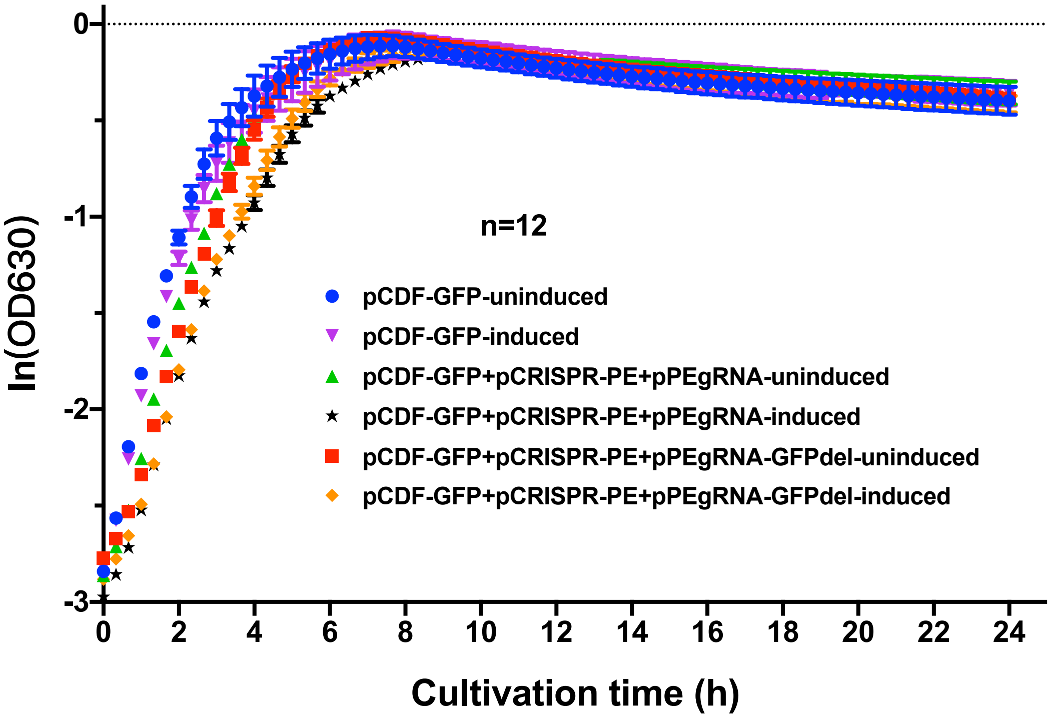

Supplementary Figure 6. Growth profiles of E. coli strains transformed with different plasmids under induced and uninduced conditions.

Growth profiling was performed in a ELx808 plate reader (Buch & Holm A/S). The 96-well microtiter plate with F-bottoms and a lid was incubated in the plate reader at 37 °C with constant shaking, and the OD630 was measured every 20 minutes for 24 hours. 200 μl of microtiter cultures with a starting OD630 of 0.05 was used. The concentration of inducer ATc is 200 ng/ml. All measurements were normalized based on the media blanks, and the mean and standard deviation of all 12 biological replicates were calculated.

Supplementary reference:

1 Anzalone, A. V. *et al.* Search-and-replace genome editing without double-strand breaks or donor DNA. *Nature* **576**, 149-157, doi:10.1038/s41586-019-1711-4 (2019).
2 Lin, Q. *et al.* Prime genome editing in rice and wheat. *Nat. Biotechnol.* **38**, 582-585, doi:10.1038/s41587-020-0455-x (2020).
3 Mali, P. *et al.* CAS9 transcriptional activators for target specificity screening and paired nickases for cooperative genome engineering. *Nat. Biotechnol.* **31**, 833-838, doi:10.1038/nbt.2675 (2013).
4 Ran, F. A. *et al.* Double nicking by RNA-guided CRISPR Cas9 for enhanced genome editing specificity. *Cell* **154**, 1380-1389, doi:10.1016/j.cell.2013.08.021 (2013).
5 Bowater, R. & Doherty, A. J. Making ends meet: repairing breaks in bacterial DNA by non-homologous end-joining. *PLoS Genet.* **2**, e8, doi:10.1371/journal.pgen.0020008 (2006).
6 Shuman, S. & Glickman, M. S. Bacterial DNA repair by non-homologous end joining. *Nat. Rev. Microbiol.* **5**, 852-861, doi:10.1038/nrmicro1768 (2007).
7 Tong, Y., Charusanti, P., Zhang, L., Weber, T. & Lee, S. Y. CRISPR-Cas9 Based Engineering of Actinomycetal Genomes. *ACS Synth. Biol.* **4**, 1020-1029, doi:10.1021/acssynbio.5b00038 (2015).
8 Qi, L. S. *et al.* Repurposing CRISPR as an RNA-guided platform for sequence-specific control of gene expression. *Cell* **152**, 1173-1183, doi:10.1016/j.cell.2013.02.022 (2013).
9 Rhodius, V. A. *et al.* Design of orthogonal genetic switches based on a crosstalk map of σs, anti-σs, and promoters. *Mol. Syst. Biol.* **9**, 702, doi:10.1038/msb.2013.58 (2013).
10 Hsu, J. Y. *et al.* PrimeDesign software for rapid and simplified design of prime editing guide RNAs. doi:10.1101/2020.05.04.077750.
