# Supplementary material for: A versatile genetic engineering toolkit for E. coli based on CRISPR-prime editing
Source: Nat Commun. 2021 Sep 1;12:5206. doi: 10.1038/s41467-021-25541-3 (PMC8410854; doi:10.1038/s41467-021-25541-3)
Supplement: Supplementary file 2 — Reporting Summary [file 41467_2021_25541_MOESM2_ESM.pdf]

nature research

Corresponding author(s): Yaojun Tong, Tilmann Weber and Sang Yup Lee

Last updated by author(s): Aug 1, 2021

Reporting Summary

Nature Research wishes to improve the reproducibility of the work that we publish. This form provides structure for consistency and transparency in reporting. For further information on Nature Research policies, see our Editorial Policies and the Editorial Policy Checklist.

Statistics

For all statistical analyses, confirm that the following items are present in the figure legend, table legend, main text, or Methods section.

n/a	Confirmed	
☐	☒	The exact sample size (*n*) for each experimental group/condition, given as a discrete number and unit of measurement
☐	☒	A statement on whether measurements were taken from distinct samples or whether the same sample was measured repeatedly
☒	☐	The statistical test(s) used AND whether they are one- or two-sided<br>*Only common tests should be described solely by name; describe more complex techniques in the Methods section.*
☒	☐	A description of all covariates tested
☒	☐	A description of any assumptions or corrections, such as tests of normality and adjustment for multiple comparisons
☐	☒	A full description of the statistical parameters including central tendency (e.g. means) or other basic estimates (e.g. regression coefficient) AND variation (e.g. standard deviation) or associated estimates of uncertainty (e.g. confidence intervals)
☒	☐	For null hypothesis testing, the test statistic (e.g. *F*, *t*, *r*) with confidence intervals, effect sizes, degrees of freedom and *P* value noted<br>*Give P values as exact values whenever suitable.*
☒	☐	For Bayesian analysis, information on the choice of priors and Markov chain Monte Carlo settings
☒	☐	For hierarchical and complex designs, identification of the appropriate level for tests and full reporting of outcomes
☒	☐	Estimates of effect sizes (e.g. Cohen's *d*, Pearson's *r*), indicating how they were calculated

Our web collection on statistics for biologists contains articles on many of the points above.

Software and code

Policy information about availability of computer code

Data collection	Sequences displayed in this study was Sanger sequenced by Eurofins Genetics.<br>Colony counts were counted using a Doc-IT imaging station from Fisher Scientific.<br>Illumina sequencing were carried out by Novogene Co., Ltd. (Beijing, China).
Data analysis	Sanger sequences were analyzed and displayed by SnapGene (V4.3.6) .<br>Illumina sequencing data was analyzed by Trim Galore (v. 0.6.4_dev, Cutadapt v. 2.10) with the switches --length 100 and --quality 20. All mutation calls were performed using breseq (v. 0.33.2, bowtie2 v. 2.3.4.1).<br>Prism (version 8) was used to plot all charts.<br>Some of the PEgRNAs were generated by PrimeDesign (https://drugthatgene.pinellolab.partners.org/)

For manuscripts utilizing custom algorithms or software that are central to the research but not yet described in published literature, software must be made available to editors and reviewers. We strongly encourage code deposition in a community repository (e.g. GitHub). See the Nature Research guidelines for submitting code & software for further information.

Data

Policy information about availability of data

All manuscripts must include a data availability statement. This statement should provide the following information, where applicable:

- Accession codes, unique identifiers, or web links for publicly available datasets
- A list of figures that have associated raw data
- A description of any restrictions on data availability

All data used in this study can be found in the manuscript and/or in the supplementary information. All Illumina sequencing data has been deposited to NCBI. Data

for on/off target evaluation: NCBI BioProject PRJNA752926; SRA accessions SRR15371474 - SRR15371494.
Data for editing escapers: NCBI BioProject PRJNA75292; SRA accessions SRR15371770 - SRR15371774.

Field-specific reporting

Please select the one below that is the best fit for your research. If you are not sure, read the appropriate sections before making your selection.

[x] Life sciences   [ ] Behavioural & social sciences   [ ] Ecological, evolutionary & environmental sciences

For a reference copy of the document with all sections, see nature.com/documents/nr-reporting-summary-flat.pdf

Life sciences study design

All studies must disclose on these points even when the disclosure is negative.

Sample size	Before knowing the system works in DNA editing of E. coli, 24 non-fluorescent clones were randomly picked for validation using Sanger sequencing; when the functionality of the CRISPR-prime editing toolkit was confirmed, we tended to randomly pick less (4-12) phenotype-positive clones for Sanger sequencing validation. Then 1-2 Sanger sequencing-positive clones for Illumina sequencing confirmation. For the analysis of "CRISPR-prime editing escapers", 10 fluorescent clones were randomly picked and sequenced.
Data exclusions	No data was excluded.
Replication	Three or more biological and technical replicates were evaluated with positive for related experiments.
Randomization	Clones for Sanger sequencing were randomly picked. 1-2 Sanger sequencing-positive clones were also randomly picked for Illumina sequencing.
Blinding	Blinding was not relevant to this study. All data were collected corresponding to the designated plasmids, phenotype, and genotype of the E. coli clones.

Reporting for specific materials, systems and methods

We require information from authors about some types of materials, experimental systems and methods used in many studies. Here, indicate whether each material, system or method listed is relevant to your study. If you are not sure if a list item applies to your research, read the appropriate section before selecting a response.

Materials & experimental systems

n/a	Involved in the study	
[x]	[ ]	Antibodies
[x]	[ ]	Eukaryotic cell lines
[x]	[ ]	Palaeontology and archaeology
[x]	[ ]	Animals and other organisms
[x]	[ ]	Human research participants
[x]	[ ]	Clinical data
[x]	[ ]	Dual use research of concern

Methods

n/a	Involved in the study	
[x]	[ ]	ChIP-seq
[x]	[ ]	Flow cytometry
[x]	[ ]	MRI-based neuroimaging
